# Dual-Band Transmissive Cross-Polarization Converter with Extremely High Polarization Conversion Ratio Using Transmitarray

**DOI:** 10.3390/ma12111827

**Published:** 2019-06-05

**Authors:** Jianxing Li, Jialin Feng, Bo Li, Hongyu Shi, Anxue Zhang, Juan Chen

**Affiliations:** 1 School of Electronic and Information Engineering, Xi’an Jiaotong University, Xi’an 710049, China; jianxingli.china@xjtu.edu.cn (J.L.); fenghui@stu.xjtu.edu.cn (J.F.); honyo.shi1987@gmail.com (H.S.); anxuezhang@xjtu.edu.cn (A.Z.); 2 Institute of Electronic Engineering, China Academy of Engineering Physics, Mianyang 621999, China; libocaepiee@163.com; 3 Shenzhen Research School, Xi’an Jiaotong University, Shenzhen 518057, China; 4 Guangdong Xi’an Jiaotong University Academy, Foshan 528300, China

**Keywords:** cross-polarization converter, transmitarray, high polarization conversion ratio

## Abstract

In this paper, a dual-band cross-polarization converter is proposed. The proposed device can convert linearly polarized incident waves to their cross-polarized transmitted waves. Inspired by the aperture coupled transmitarray, a transmissive multi-layered unit cell structure was designed, which can operate in two frequency bands. The designed structure can manipulate the polarization of the transmitted wave into the cross-polarization of the incident waves at 10.36 GHz and 11.62 GHz. The cross-polarized transmittance of the proposed cross-polarization converter is higher than 0.93. In addition, the transmitted wave has an extremely low co-polarized component, which results in a nearly 100% polarization conversion ratio. The two working frequencies can be tuned independently. The proposed cross-polarization converter was simulated, fabricated and measured. The simulation results confirm with the measurement results.

## 1. Introduction

Manipulations of microwave properties have attracted great interest due to their applications for wireless communication [1,2], imaging [3,4,5], stealth technology [6], etc. Techniques using artificial structures, artificial materials or metamaterials have been applied for controlling the polarization [7], amplitude [8], beam shape [9] and direction [10] of microwave. Polarization is one of the basic and important properties of microwaves and has an impact on the performance of communication or radar systems. Polarization converters can be applied for antenna design in microwave communication, remote sensing and imaging systems [11,12,13]. For instance, by converting a horizontally polarized radar antenna to a vertically polarized radar antenna, a polarization converter can reduce the influences of the ground or sea clutter. In addition, for frequency hopping radar, dual-band and multi-band polarization converter is desired. Polarization of microwaves is usually manipulated by anisotropic structures or artificial materials [14]. Reflective polarization converters are usually thin and wideband, e.g., wideband polarization converters using plasmonic hybridizations [15,16].

The transmissive cross-polarization converters are usually multi-layered artificial structures with complex metallic structures on each layer [17,18]. A single band high efficiency transmissive ultrathin cross-polariztion converter with low in-band co-polarization component was designed using anisotropic artificial structures [19]. Bi-layered chiral metamaterials were also applied for cross-polarization converters from microwave band to terahertz band. However these designs have a co-polarized component of the transmission higher than 0.2 [20,21,22,23]. Multi-layered anisotropic metasurface were also used for cross-polarized converter designs, which also has a co-polarized component of the transmitted wave of around 0.2 [24,25]. Multi-layered anisotropic metasurface with metallic gratings can be used for achieving broadband properties. However, the co-polarized component of the transmitted wave can be as high as about 0.1 [26,27]. A dual-band transmissive cross-polarization converter was designed using planar-dipole pair with a co-polarized component of abour 0.15 [28]. Cascaded cavities was used for wideband cross-polarization conversion. However, the co-polarized component is about 0.3 [29].

Reflectarrays or transmitarrays are also used for polarization conversion [30,31,32,33,34]. Reflectarrays were applied for cross-polarization conversion in microwave band and near-infrared band with a co-polarized component of about 0.2 and 0.1, respectively [30,32]. Transmitarrays were applied for single band linear-to-circular polarization conversion [33,34]. Thus, dual-band transmissive cross-polarization converters with extremely low co-polarized component are still in desire.

In this paper, we combined the concepts of transmitarray and polarization converter together and proposed a transmitarray inspired dual-band transmissive cross-polarization converter with extremely low co-polarized component through the whole frequency range. Thus, it can block power transmission in the out-of-band and results in a better frequency selective characteristic. The designed dual-band transmissive cross-polarization converter can convert a linear polarized incident wave to its cross-polarized wave at 10.36 GHz and 11.62 GHz. At these two frequencies, the cross-polarized transmittances are higher than 0.93 and the co-polarized transmittances are suppressed to be 0.0047 and 0.0043, which leads to an almost 100% polarization conversion ratio. In addition, the operating frequencies can be separately tuned by changing the design parameters, which makes this design more useful and general for different practical applications. To our knowledge, it is the first time that the concept of transmitarray is applied for transmissive dual-band cross-polarization converter.

## 2. Dual-Band Cross-Polarized Converter Design

**Figure 1:**
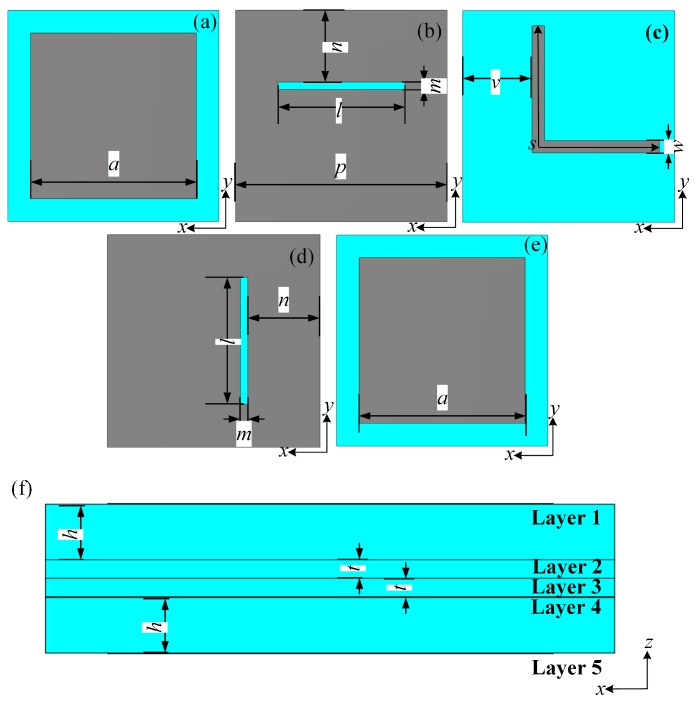
Geometry of the unit cell: (**a**) Layer-1. (**b**) Layer-2. (**c**) Layer-3. (**d**) Layer-4. (**e**) Layer-5. (**f**) Side view.

The proposed cross-polarization converter is a five-layered structure. Such structure is inspired from patch antenna based transmitarray. Different to the previously designed multi layered devices in which all layers contribute to the resonance, only top and bottom layers of the proposed cross-polarization converter resonate. Thus, the insert loss caused by resonances is significantly reduced, which leads to a relatively high efficiency. Structures in each layer of the proposed device unit cell are shown in Figure 1. The blue part and gray part in Figure 1 are substrate and metallic sheet, respectively. The layer-1 and layer-5 are patch elements, which couple and decouple the incident electromagnetic (EM) wave, respectively. These patch elements can be considered as slot coupled square patch antennas. The layer-2 and layer-4 are slots used for wave coupling. Together with layer-2 and layer-4, the layer-3 can be considered as a stripline with a total length *s*, which contributes to the cross-polarization conversion in the unit cell and can give an additional transmission phase.

As shown in Figure 1a, the structure on layer-1 is a square patch that is fed by a rectangular slot along the *x*-axis on the layer-2 shown in Figure 1b. The square patch on layer-1 couples the *y*-polarized incident wave into the unit cell. The incident wave is then coupled to an “L” shaped metallic line on layer-3 through the slot on layer-2. The “L” shaped metallic line is shown in Figure 1c. Such an “L” shaped metallic line transforms the wave propagation along the *y*-axis into wave propagation along the *x*-axis. Then, through the rectangular slot along the *y*-axis on the layer-4 shown in Figure 1d, the waves in the “L” shaped metallic line is coupled to the square patch on layer-5. The layer-5 is the same with the layer-1 as shown in Figure 1e. Layer-5 can decouple the incident wave to *x*-polarized transmitted wave. Figure 1f shows the side view of the unit cell. Thus, only the layer-1 and layer-5 are resonant structures which help increase the transmission efficiency. The geometric parameters are selected as a=11 mm, m=5 mm, n=4.65 mm, p=14 mm, v=4.6 mm, l=8.4 mm, w=0.8 mm, m=0.7 mm, h=1.5248 mm, t=0.508 mm and s=16 mm. The dielectric is Taconic TLY-5 with a permittivity of 2.2 and a loss tangent of 0.0009.

## 3. Simulation Results

The unit cell model was built up and simulated in a commercial software CST MICROWAVE STUDIO. In the simulation, the unit cell boundary condition was used along the *x*- and *y*-directions, and the absorbing boundary condition was applied for the *z*-direction. The unit cell model was excited by Flouquet ports with a unit normal incidence of linearly polarized waves along *y*-axis in the frequency range from 10 GHz to 12 GHz. The amplitude of the transmittances is represented by T. T${}_{\mathrm{co}}$ is the co-polarized transmittance of the transmitted wave. T${}_{\mathrm{cr}}$ refers to the cross-polarized transmittance of the transmitted wave. The reflectance is presented by R.

**Figure 2:**
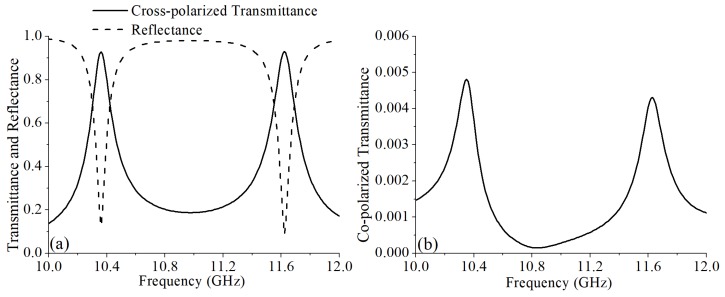
(**a**) The simulated cross-polarized transmittance and reflectance. (**b**) The simulated co-polarized transmittance.

**Figure 3:**
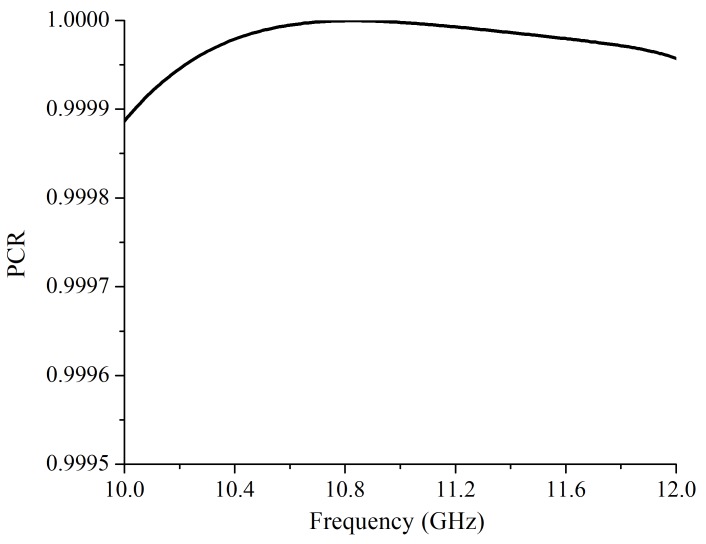
The simulated polarization conversion ratio.

Figure 2a shows the simulation results of T${}_{\mathrm{cr}}$ and R with an incidence wave propagating along *z*-axis. The simulation results show that the designed structures can work at 10.36 GHz and 11.62 GHz. At 10.36 GHz, the T${}_{\mathrm{cr}}$ is 0.93, and the reflectance is 0.126. At 11.62 GHz, the T${}_{\mathrm{cr}}$ is 0.931, and the reflectance is 0.088. When the T${}_{\mathrm{cr}}$ reach the maximum, the reflectance is the minimum. The T${}_{\mathrm{co}}$ is a key parameter of a cross-polarization converter that decides the polarization conversion ratio (PCR). The PCR is defined as $T_{\mathrm{cr}}^{2}/\left(T_{\mathrm{co}}^{2}+T_{\mathrm{cr}}^{2}\right)$ and can directly reflect the polarization purity of the transmitted wave. The simulation result of the T${}_{\mathrm{co}}$ is shown in Figure 2b. The T${}_{\mathrm{co}}$ is 0.0047 and 0.0043 at 10.36 GHz and 11.62 GHz, respectively. Thus, according to the numerical data, the PCR was nearly 100% at 10.36 GHz and 11.62 GHz, which demonstrates that this design can obtain an extremely low T${}_{\mathrm{co}}$ and an extremely high PCR as shown in Figure 3. Thus, the transmitted wave has an extremely high polarization purity.

## 4. Discussion

**Figure 4:**
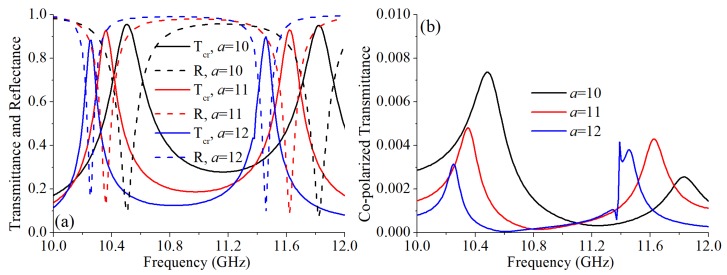
Simulation results with different *a*: (**a**) The simulated cross-polarized transmittance and reflectance. (**b**) The simulated co-polarized transmittance.

**Figure 5:**
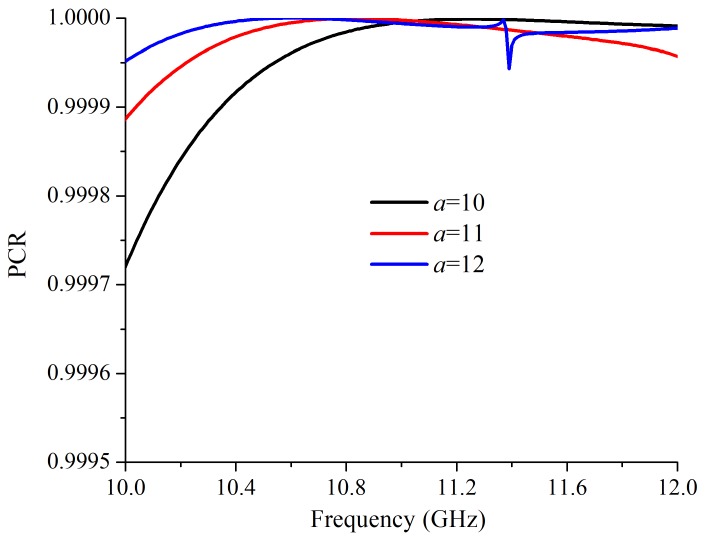
The simulated polarization conversion ratios with different *a*.

**Figure 6:**
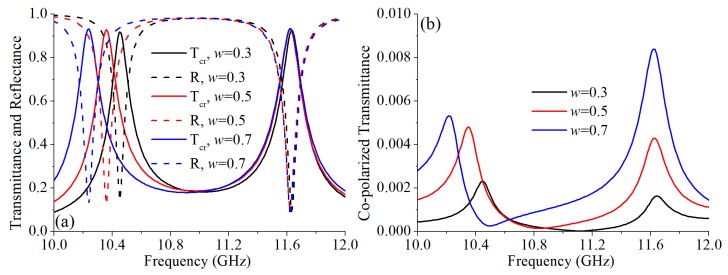
Simulation results with different *w*: (**a**) The simulated cross-polarized transmittance and reflectance. (**b**) The simulated co-polarized transmittance.

**Figure 7:**
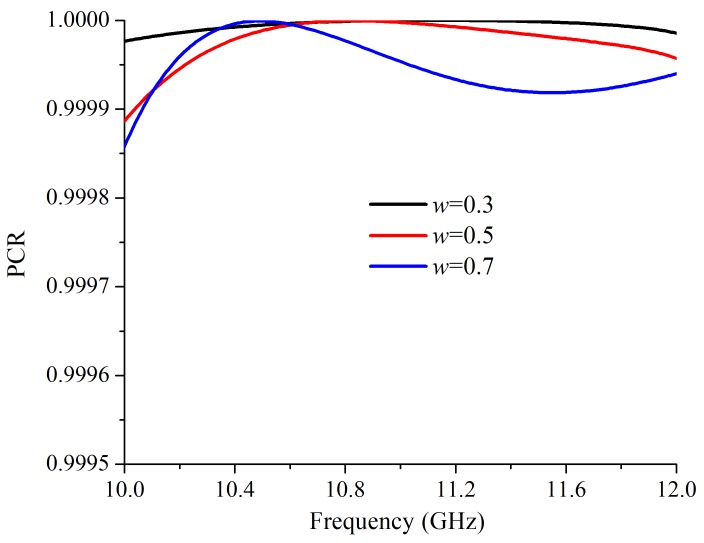
The simulated polarization conversion ratios with different *w*.

To provide an insight into the different design parameters, and to illustrate their influence on the frequency behaviour of the proposed cross-polarized converter, parameter sweeps for various *a* and *w* were analyzed by simulations. *a* and *w* are the key parameters that have significant impacts on the frequency behaviour of the cross-polarized converter. The influences of *a* are shown in Figure 4 and Figure 5. The influences of *w* are shown in Figure 6 and Figure 7.

As shown in Figure 4a, with a larger *a*, the two operating frequencies had a red shift. When *a* was 10 mm and 12 mm, the working frequency of the cross-polarized converter were 10.5 GHz/11.82 GHz and 10.26 GHz/11.46 GHz, respectively. In addition, with a larger *a*, the cross-polarized transmission peak was higher. The co-polarized transmittances with *a* = 10 mm and *a* = 12 mm are shown in Figure 4b. When *a* = 10 mm, the T${}_{\mathrm{co}}$ at 10.5 GHz and 11.82 GHz are 0.0073 and 0.0025, respectively. At 10.26 GHz and 11.46 GHz, with *a* = 12 mm, the T${}_{\mathrm{co}}$ are 0.0031 and 0.0038, respectively. The calculated PCRs with different *a* are shown in Figure 5. When *a* changed, the PCR maintained nearly 100%.

The parameter *w* was another key parameter that impacted on the working frequency. Different to *a*, *w* only influence the lower frequency. As shown in Figure 6a, with a larger *w*, the lower operating frequency has a red shift. When *w* is 0.3 mm and 0.7 mm, the lower working frequency of the cross-polarized converter are 10.45 GHz and 10.24 GHz, respectively. However, when *w* changed, the higher working frequency almost unchanged. Thus, by varying *a* and *w*, the two working frequencies can be tuned independently, which enhances the practicability of the proposed cross-polarization converter. In addition, with a larger *w*, the cross-polarized transmission peak is slightly higher. The co-polarized transmittances with different *w* are shown in Figure 4b. When *w* = 0.3 mm, the T${}_{\mathrm{co}}$ at 10.45 GHz and 11.62 GHz were 0.0023 and 0.0016, respectively. At 10.24 GHz and 11.62 GHz, with *w* = 0.7 mm, the T${}_{\mathrm{co}}$ are 0.005 and 0.0084, respectively. The T${}_{\mathrm{co}}$ was higher with a larger *w*. The calculated PCRs with different *w* are shown in Figure 5. When *w* changed, the PCR maintains nearly 100%.

**Figure 8:**
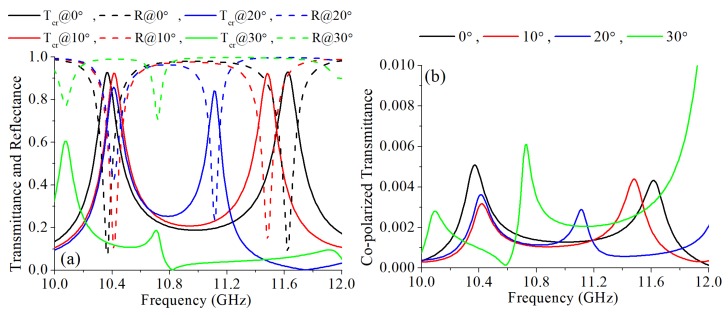
Simulation results with different incident angles: (**a**) The simulated cross-polarized transmittance and reflectance. (**b**) The simulated co-polarized transmittance.

To further explore the properties of the proposed design under different incident angles, additional simulations were done with incident angles of 10${}^{\circ }$, 20${}^{\circ }$ and 30${}^{\circ }$. The simulation results are shown in Figure 8. As shown in Figure 8a, with a larger incident angle within 20${}^{\circ }$, the lower transmission frequency maintains with a little fluctuation and the higher transmission frequency has a red shift. When the incident angle increased to 30${}^{\circ }$, the cross-polarization converter is invalidated. The co-polarized component of the transmitted wave maintains extremely low under these incident angles, as shown in Figure 8b. Although the property of the proposed design under oblique incidence is not outstanding, it still can be applied for applications where the polarization converter used with a fixed exciting antenna.

## 5. Measurement Results

**Figure 9:**
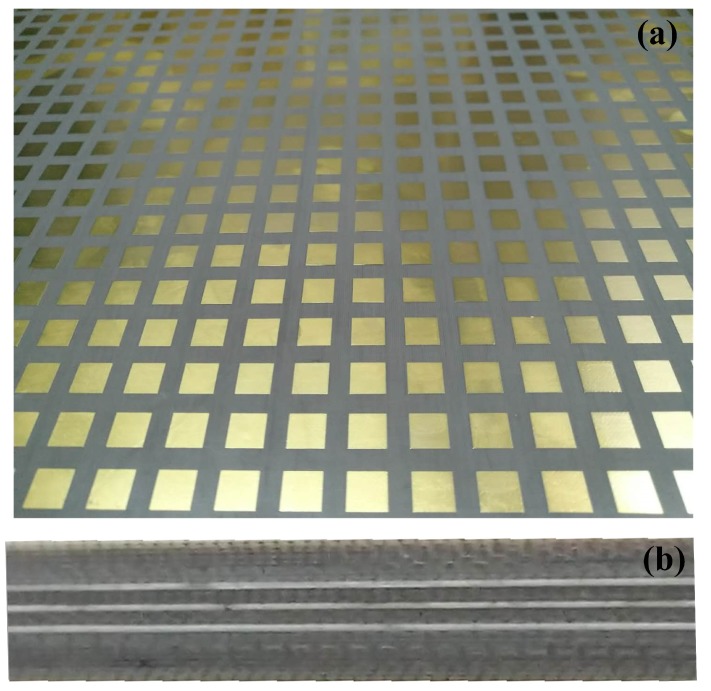
The fabricated sample of the cross-polarized converter: (**a**) Top view of the sample. (**b**) Side view of the sample.

The above proposed design was fabricated by printed circuit board (PCB) processing. The dimension of the sample was 308 mm × 224 mm containing 22 × 16 unit cells. Because the layer-1 and layer-5 are the same, only top view of the fabricated sample is shown in Figure 9.

**Figure 10:**
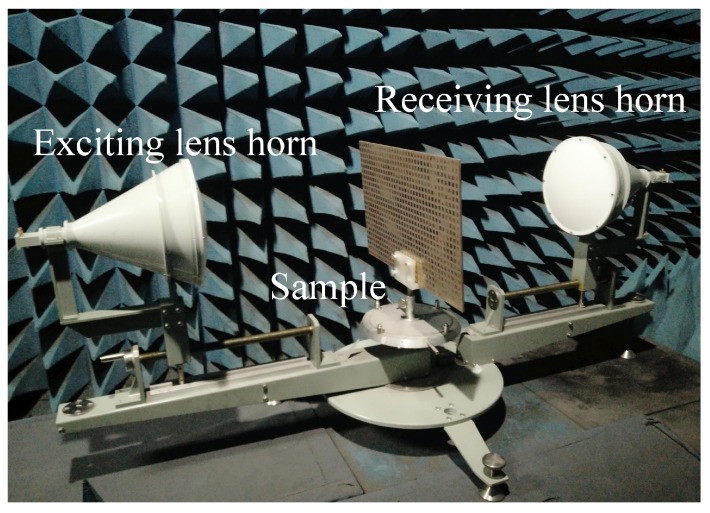
Photo of the measurement setup.

**Figure 11:**
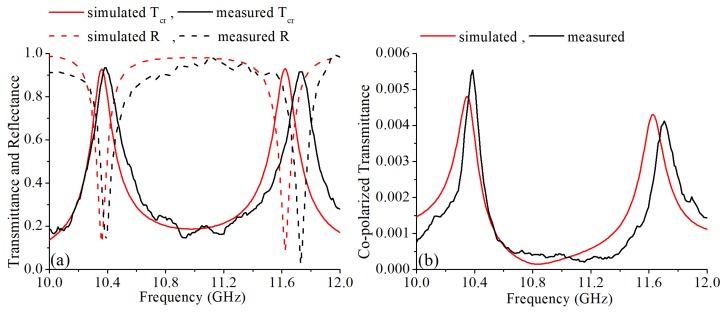
(**a**) The measured and simulated cross-polarized transmittances and reflectances. (**b**) The measured and simulated co-polarized transmittances.

**Figure 12:**
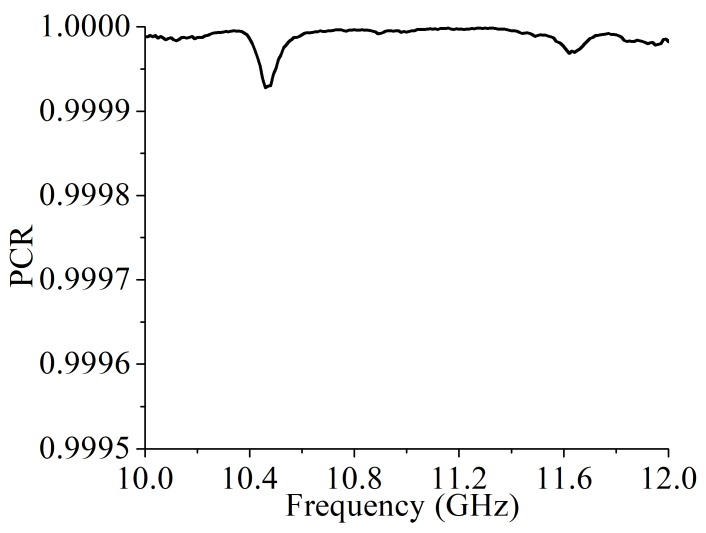
The polarization conversion ratio calculated from the measured results.

The measurement setup is shown in Figure 10. Two spot focusing lens horns were used as exciting and receiving antennas. The transmittances and the reflectance were measured by a vector network analyzer (Agilent E8363b). The measured transmittances are shown in Figure 11. The measurement results confirm with the simulated results and their differences are caused by the machining errors, measurement errors and the background noise. The transmittances of the cross-polarization converter sample are shown in Figure 11a. The measured cross-polarized transmittances were 0.935 and 0.914 at 10.37 GHz and 11.71 GHz, respectively. The measured reflectance at these two frequency bands were 0.138 and 0.025, respectively. At 10.37 GHz and 11.71 GHz, the measured co-polarized transmittance was significantly suppressed and was below 0.005 as shown in Figure 11b. The PCR calculated from the measured results is nearly 100% as shown in Figure 12. Thus, the fabricated cross-polarized converter had high efficiency for both transmitted power and polarization conversion.

## 6. Conclusions

In conclusion, a dual-band high PCR cross-polarization converter is designed, fabricated and measured. This design is inspired by the concept of transmitarray. The cross-polarized transmittance is higher than 0.9 at 10.37 GHz and 11.71 GHz. The co-polarized transmittance is significantly suppressed below 0.005, which leads to an almost perfect PCR. Thus, the transmitted wave has an extremely high polarization purity. In addition, by varying *a* and *w*, the two working frequencies can be tuned independently. The presented design represents a basis for the development of transmissive metasurfaces for wavefront control by changing the length of the stripline on layer-3. The proposed design also can be used for transmissive linear-to-circular polarization converter by using a truncated square patch on the layer-5 instead. The proposed cross-polarization converter can be employed in antenna, radar or telecommunication applications.
